# Baseline and dynamic changes in skeletal muscle mass as predictive biomarkers in patients with metastatic renal cell carcinoma treated with Nivolumab

**DOI:** 10.2478/raon-2025-0065

**Published:** 2025-12-16

**Authors:** Erdem Ozkan, Murathan Koksal, Bunyamin Ece, Mustafa Koyun, Omer Faruk Kuzu, Yusuf Acikgoz, Efnan Algin

**Affiliations:** 1 Radiology Department, Kastamonu Training and Research Hospital, Kastamonu, Türkiye; 2 Radiology Department, Ankara Bilkent City Hospital, Ankara, Türkiye; 3 Radiology Department, Kastamonu University, Faculty of Medicine, Kastamonu, Türkiye; 4 Department of Medical Oncology, Çankırı State Hospital, Çankırı, Türkiye; 5 Department of Medical Oncology, Lokman Hekim University Ankara Hospital, Ankara, Türkiye; 6 Department of Medical Oncology, Ankara Bilkent City Hospital, Ankara, Türkiye

**Keywords:** renal cell carcinoma, low muscle mass, computed tomography, nivolumab

## Abstract

**Background:**

Low skeletal muscle mass has been increasingly recognized as a negative prognostic factor in oncology. According to the European Working Group on Sarcopenia in Older People 2 (EWGSOP2), sarcopenia is defined as a progressive and generalized skeletal muscle disorder characterized by the loss of muscle strength and muscle mass, which can lead to impaired physical performance. This study aimed to investigate whether baseline low muscle mass and dynamic changes in muscle mass during immunotherapy could predict treatment response and survival in patients with metastatic renal cell carcinoma (mRCC) treated with Nivolumab.

**Patients and methods:**

This retrospective cohort study included 50 mRCC patients (35 men, 15 women; mean age 59.1 ± 10.2 years) who received Nivolumab between 2019 and 2022 and underwent abdominal computed tomography (CT) before and during treatment. Muscle mass was assessed by calculating the skeletal muscle index (SMI) at the third lumbar vertebra using standard Hounsfield unit thresholds (-29 to +150 HU). Treatment response was evaluated according to immune Response Evaluation Criteria in Solid Tumors (iRECIST). Overall survival (OS) and progression-free survival (PFS) were analyzed using Kaplan–Meier curves and Cox regression models.

**Results:**

Low muscle mass was identified in 60% of patients and was significantly associated with multiple organ metastases (p = 0.003). Patients with baseline low muscle mass or a negative change in SMI during treatment demonstrated poorer treatment response (p = 0.027 and p = 0.021, respectively). Both OS and PFS were significantly shorter in patients with low muscle mass and those with declining muscle mass during treatment.

**Conclusions:**

Pre-treatment low muscle mass and muscle mass decline during immunotherapy were independently associated with inferior survival and treatment response in mRCC patients receiving Nivolumab. CT-based muscle mass assessment may serve as an imaging-based prognostic biomarker in this population.

## Introduction

Kidney cancer accounts for approximately 5% of all new cancer diagnoses in men and 3% in women worldwide.^1^ According to Global Cancer Observatory (GLOBOCAN) 2020 estimates, renal cell carcinoma (RCC) ranks as the 14^th^ most commonly diagnosed cancer globally and represents more than 85% of all primary renal malignancies.^1,2^ Furthermore, updated GLOBOCAN data reveal that in 2022, approximately 20 million new cancer cases and 9.7 million cancer-related deaths occurred worldwide, reflecting the growing global burden of malignancies.^3^ At initial diagnosis, approximately 70% of patients have localized disease in the kidney, while the remaining 30% present with regional or distant organ metastases.^4^ The treatment of renal malignancies differs between localized and metastatic disease; standard treatments such as partial or radical nephrectomy are applied for localized disease, whereas in advanced or metastatic cases, in addition to cytoreductive nephrectomy, “targeted” therapies, cytokine treatments, and immunotherapy can be utilized. Significant advancements have been made in the treatment of metastatic renal cell carcinoma (mRCC), particularly in recent years. Tyrosine kinase inhibitors targeting vascular endothelial growth factor, platelet derived growth factor receptor, MET, AXL and immunotherapeutic agents effective at immune checkpoint inhibition have emerged as the most prominent treatment modalities.^5^ Nivolumab, an immunotherapeutic agent, is the first immune checkpoint inhibitor approved for the treatment of mRCC, developed against the programmed death-1 (PD-1) antigen.^6^ Nivolumab prevents the programmed death ligand-1 molecule on tumor cells from binding to the PD-1 molecule on T cells, thus facilitating the activation of T cells against tumor cells in RCC with single or multiple metastases. However, as with many treatment modalities, there are numerous factors influencing the treatment response to Nivolumab. These factors include tumor burden, tumor subtype, type of mutation, cancer stage, number and location of metastatic organs, patient performance status, and comorbidities.^6^

According to the European Working Group on Sarcopenia in Older People 2 (EWGSOP2), sarcopenia is defined as a progressive and generalized skeletal muscle disorder characterized by the loss of muscle strength and muscle mass, which can lead to impaired physical performance.^7^ Low muscle strength is considered the most important and primary indicator of sarcopenia, with ‘confirmed sarcopenia’ diagnosed when low muscle strength is accompanied by low muscle quantity or quality.^7^ However, in oncological research, CT-based assessment of skeletal muscle mass has been widely used as a surrogate marker for overall muscle status due to its availability in routine clinical practice. Low skeletal muscle mass, as measured by CT, represents a key component of cancer cachexia and has been consistently associated with poor clinical outcomes. The rate of sarcopenia in patients with localized RCC is reported to be approximately 47%, while in mRCC patients, this rate has been indicated to vary between 29% and 68%.^8,9,10^ The presence or absence of low muscle mass is considered one of the prognostic factors in assessing treatment response and tolerance in mRCC, as well as in overall survival (OS) and progression-free survival (PFS).^10^

In the evaluation of low muscle mass in cancer patients, computed tomography (CT) is considered the gold standard imaging modality, as cross-sectional muscle measurements, particularly at the third lumbar vertebra (L3), provide validated surrogate markers of total body muscle mass.^7^ In patients with non-cancerous conditions or in healthy populations, alternative methods such as bioelectrical impedance analysis or dual-energy X-ray absorptiometry are often preferred due to the high radiation exposure associated with CT imaging.^11^ The Skeletal Muscle Index (SMI), derived from the ratio of the cross-sectional area of skeletal muscle (SMA) measured from a single CT slice to the square of the height in meters, is the most commonly used parameter in the assessment of muscle mass.^12^

While previous studies have established that low muscle mass is associated with poor outcomes in mRCC patients, several gaps remain in the literature. First, most studies have focused on baseline muscle assessment without evaluating dynamic changes during treatment. Second, the use of immune Response Evaluation Criteria in Solid Tumors (iRECIST) for response evaluation in the context of muscle mass assessment has been underexplored. Third, the predictive value of muscle mass changes specifically during nivolumab therapy, as opposed to other systemic treatments, requires further investigation given the unique mechanisms of immunotherapy.

The aim of this study is to conduct quantitative muscle mass analyses based on CT examinations obtained at baseline and during the 6th to 12th weeks of Nivolumab treatment in patients with mRCC. We aim to evaluate the effect of pre-treatment low muscle mass on OS, PFS, and treatment response. Additionally, we aimed to determine the impact of dynamic changes in muscle mass during the treatment process on OS, PFS, and treatment response - an aspect that has received limited attention in the immunotherapy literature.

The inclusion of patients treated with Nivolumab, an immunotherapeutic agent, and the use of ‘‘immune Response Evaluation Criteria in Solid Tumours’’ (iRECIST) criteria for response evaluation distinguish our study from similar research in the literature.^13^ This methodological choice is particularly important given the potential for pseudoprogression, a transient increase in tumor burden caused by immune cell infiltration, which is frequently observed during immunotherapy. In addition, the evaluation of changes in SMI during treatment and their association with both treatment response and survival represents another key strength of our study.

## Patients and methods

### Patients

**FIGURE 1.**
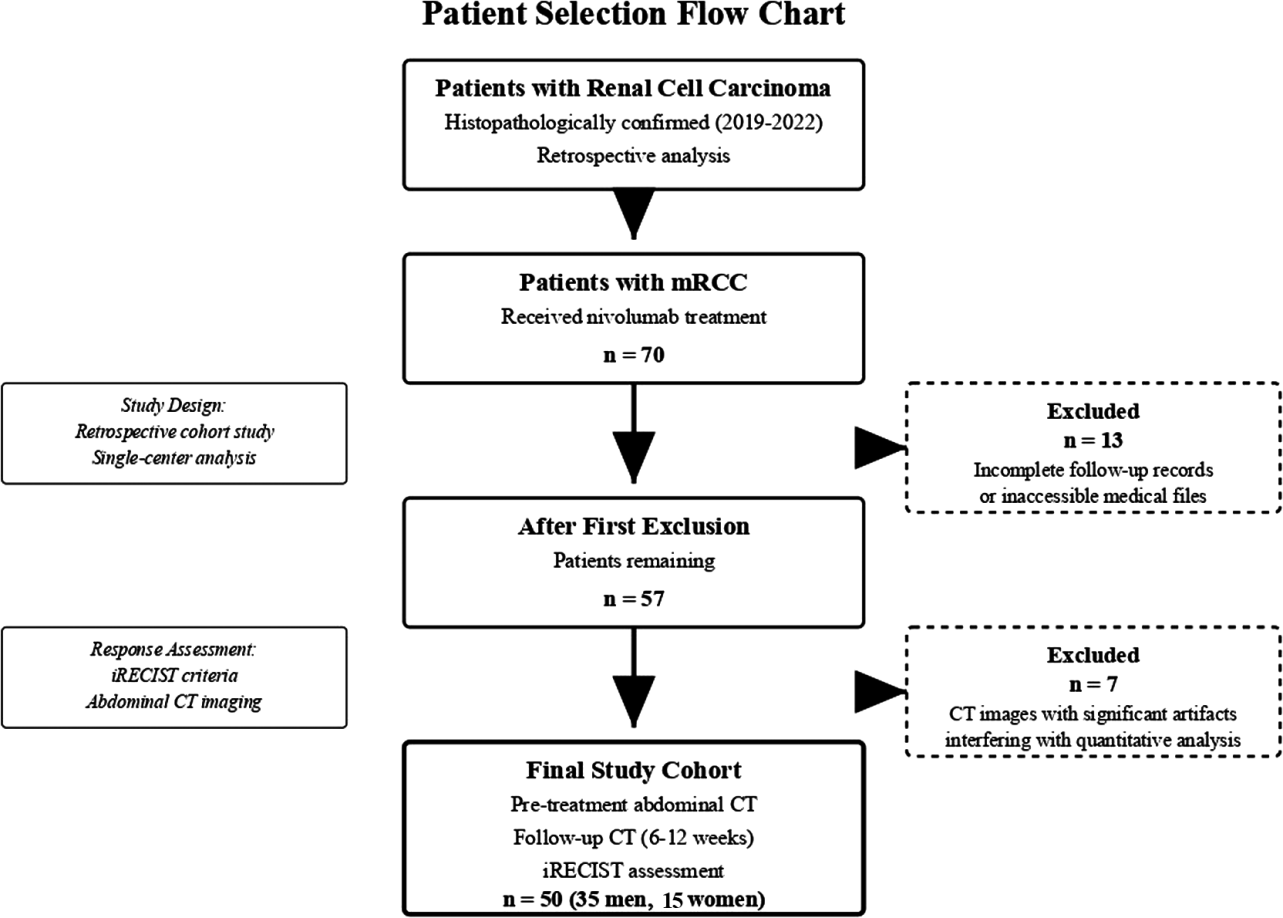
Patient selection flow chart for the retrospective analysis of nivolumab treatment in metastatic renal cell carcinoma.

The present retrospective cohort study received approval from the Clinical Research Ethics Committee of our hospital (Decision No: E1-22-2532, April 6, 2022) prior to its initiation. Patients diagnosed with RCC histopathologically at our hospital between 2019 and 2022 were retrospectively analyzed. Of those, 70 patients with mRCC and received Nivolumab for mRCC were identified as eligible for the study. 13 patients with incomplete follow-up records or inaccessible files were excluded from the study group. 7 patients whose CT images exhibited significant artifacts that interfered with accurate interpretation and caused errors in quantitative analysis were excluded from the study (Figure 1). Consequently, 50 patients (35 men, 15 women) who underwent abdominal CT imaging prior to nivolumab treatment and follow-up abdominal CT scans within 6 to 12 weeks for response assessment based on the iRECIST were included in the final cohort. Demographic characteristics including age and sex distribution were recorded for all patients. Age data are reported as mean ± standard deviation, and categorical variables as frequencies and percentages. Immunotherapy agents may elicit unconventional tumour responses, such as pseudoprogression, which challenge standard assessment criteria. The iRECIST guideline provides a standardized framework to capture and classify these atypical patterns, improving the accuracy of response evaluation in immunotherapy trials. Therefore, control CT scans obtained 6 to 12 weeks after treatment were reviewed and patients’ responses to immunotherapy were assessed according to iRECIST criteria. The patients’ sex, age at the time of RCC initial diagnosis, method of diagnosis (biopsy, nephrectomy), whether they underwent surgery, stage at diagnosis, histopathological type of RCC, Fuhrman grade, ‘International Metastatic RCC Database Consortium’ (IMDC) score and risk group, metastasis characteristics (lymph node metastasis, distant organ metastasis, combination of lymph node and distant organ metastases, etc.), the date of initiation of Nivolumab treatment, and whether progression occurred under nivolumab were recorded. The OS and PFS outcomes of the patients were calculated.

### CT protocol and image analysis

**FIGURE 2.**
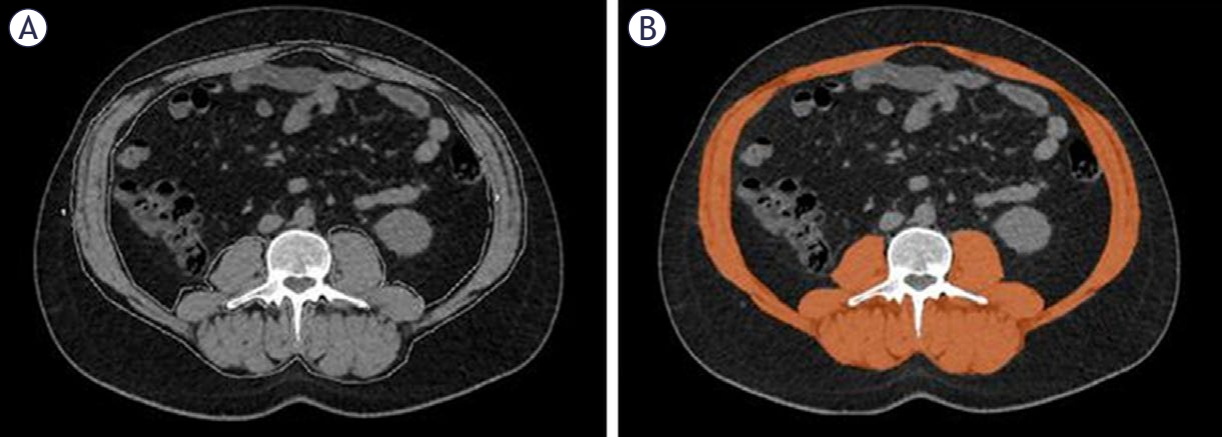
Axial computed tomography images at the level of the third lumbar vertebra. **(A)** Unprocessed image used for skeletal muscle area analysis. **(B)** Skeletal muscle areas (highlighted in orange) were manually segmented based on a Hounsfield unit (HU) threshold range of -29 to +150 to quantify muscle tissue area.

Abdominal CT examinations were obtained using a 128-slice multidetector CT scanner (General Electric Revolution Evo 128, Milwaukee, USA). The technical parameters used in the scan protocol in both CT scans of the patients were as follows: 2 mm collimation, 2 mm slice thickness, rotation time: 0.6 seconds, pitch: 1, FOV: 40 cm, kV: 120, mA: 200-400. Two radiologists (15 and 5 years of experience with abdominal CT) evaluated the cases independently and blinded to clinical notes and laboratory and radiological reports in the picture archiving and communication systems (PACS). A specialized software program (Advantage Workstation 4.7 Revolution, General Electric, Milwaukee, USA) was used for the quantitative assessment of muscle mass. In the abdominal CT scans obtained before the initiation of nivolumab treatment, skeletal muscles at the L3 vertebral level (rectus abdominis, lateral and oblique abdominal muscles, psoas major, quadratus lumborum, erector spinae, and multifidus muscles) were evaluated from a single slice. To identify muscle structures, a density range of (-29)/(150) Hounsfield unit, widely accepted in the literature, was selected.^14^ The area of the muscle structures in the determined density range at the L3 vertebral level was measured in square centimeters (cm^2^) and the SMA value was found (Figure 2). To normalize the measurements, the SMA was divided by the square of the patient’s height, resulting in the SMI expressed in cm^2^/m^2^. SMI was considered a continuous variable and was used as an indicator of total body muscle mass based on studies indicating that the total cross-sectional area of skeletal muscle at the L3 vertebral level is linearly associated with total body muscle mass.^15,16^ The cutoff values for SMI used to determine the presence or absence of low muscle mass were derived from previous studies conducted in similar populations, with values below 52.4 cm^2^/m^2^ in men and 38.5 cm^2^/m^2^ in women being considered as low muscle mass.^17,18^ In the follow-up abdominal CT scans performed for treatment response assessment, SMI values were re-measured from the same anatomical slices, and changes in muscle mass-related parameters were recorded throughout the treatment period. Additionally, the difference between pre-treatment SMI and post-treatment SMI was evaluated to investigate its relationship with patients’ PFS, OS, and objective response. Furthermore, patients’ treatment responses were assessed according to iRECIST criteria, and the statistical relationship with muscle mass was analyzed. Changes in SMI, denoted as ΔSMI, were calculated by subtracting the SMI value measured on CT scans obtained before the initiation of Nivolumab treatment from the value measured on control CT scans performed for treatment response evaluation. A positive ΔSMI indicated an increase in muscle mass during treatment, whereas a negative ΔSMI indicated a decrease.

### Statistical analysis and statistical power analysis

Statistical analysis was performed using SPSS 22 (Statistical Package for the Social Sciences Version 22.0 for Windows) (SPSS Inc., Chicago, IL, USA). Sex distribution within muscle mass groups was analyzed using Chi-square test. Age differences between groups were compared using independent t-test for normally distributed data or Mann-Whitney U test for non-normally distributed data, with results reported as mean ± standard deviation or median (range) as appropriate. The Chi-square test or Fisher’s exact test was used for the comparison of categorical variables, as appropriate. Univariate and multivariate analyses for OS were conducted using the Cox regression model. Survival analyses were performed using the Kaplan-Meier method, and the results were analyzed with the Log-rank test. OS was defined as the time from the start of nivolumab treatment to death or the last follow-up date for living patients. PFS was defined as the time from the start of nivolumab treatment to progression.

To assess interobserver agreement for SMA measurements, the intraclass correlation coefficient (ICC) was calculated with 95% confidence intervals. ICC values were interpreted as follows: < 0.50 indicated poor agreement, 0.50–0.75 moderate agreement, 0.75–0.90 good agreement, and 0.90–1.00 excellent agreement. Test-retest reliability was evaluated by calculating ICC between measurements obtained from CT scans performed at different time points, using the same interpretation criteria as interobserver agreement. Statistical significance was set at p < 0.05 with 95% confidence intervals.

Post hoc power analysis was conducted to evaluate the study’s ability to detect clinically meaningful differences between groups. Power calculations were performed using G*Power 3.1.9.7 and R software (version 4.3.0), based on observed effect sizes, event rates, and sample sizes. For survival endpoints, power was estimated using the logrank test and Cox proportional hazards models; for categorical outcomes, Chi-square tests and logistic regression models were used. All calculations assumed a two-sided alpha level of 0.05.

The study achieved adequate power (> 75%) for the primary survival comparisons between low muscle mass and normal muscle mass groups (85.2% for overall survival; 78.6% for progression-free survival). Multivariate Cox regression modeling also demonstrated high power (89.6%) for detecting combined effects of low muscle mass, ΔSMI, and metastatic burden on overall survival. Power for secondary endpoints, particularly treatment response analyses, ranged between 68.9% and 74.3%, which is considered acceptable for exploratory purposes. Considering the retrospective design, no prior sample size calculation was performed.

## Results

Interobserver agreement for SMA measurements was assessed using a two-way mixed-effects model with absolute agreement for single measurements ICC(2,1). The analysis demonstrated excellent agreement between the two radiologists (ICC = 0.947, p < 0.001).

### Characteristics of patients

The study included 50 patients (35 men [70%], 15 women [30%]) with a mean age of 59.1 ± 10.2 years. The mean age was 59.9 ± 9.5 years in males and 57.1 ± 11.8 years in females. Histopathologically, only 4 patients (8%) had papillary type RCC, while 46 (92%) had clear cell type RCC. IMDC score of 5 patients could not be calculated due to missing parameters. In 45 patients for whom IMDC data were available, 36 patients (80%) were classified as good risk, 5 patients (11%) as moderate risk and 4 patients (9%) as poor risk according to risk categories. At initial diagnosis, 20 patients (40%) had no metastasis, while metastasis was present in 30 patients (60%). All patients received at least one line of systemic therapy, including interferon, pazo-panib, sunitinib or everolimus, prior to nivolumab, and received nivolumab after failure of these therapies. First-line treatments included interferon in 27 patients (54%), sunitinib in 12 patients (24%), and pazopanib in 11 patients (22%). Nivolumab was administered as second-line therapy in 18 patients (36%), third-line therapy in 17 patients (34%), fourth-line therapy in 12 patients (24%), and fifthline therapy in 3 patients (6%).

In the quantitative analyses obtained from pre-treatment CT scans, low muscle mass was present in 30 patients (60%) of the entire patient group, whereas 20 patients (40%) had normal muscle mass. Sex distribution showed a difference between muscle mass groups: in the low muscle mass group, 25 patients (83.3%) were male and 5 (16.7%) were female, while in the normal muscle mass group, 10 patients (50%) were male and 10 (50%) were female (p = 0.013). The mean age in the low muscle mass group was 60.0 ± 10.5 years, while the mean age in the normal muscle mass group was 57.7 ± 9.9 years, which was not a statistically significant difference (p = 0.382).

When evaluated with respect to the organs involved in metastasis, no statistically significant association was found between low muscle mass and specific metastasis locations. However, the presence of low muscle mass was found to be statistically significantly higher in patients with multiple organ metastases (p = 0.003).

In the group with a negative change in SMI (ΔSMI-negative), the mean age was 61.0 ± 10.1 years, while in the group with a positive change (ΔSMI-positive), it was 57.1 ± 10.1 years. The mean age was higher in the ΔSMI-negative group, though this difference was not statistically significant (p = 0.180). No significant association was observed between sex and ΔSMI groups (p = 0.758). Additionally, no significant statistical relationships were identified with respect to IMDC categories, the type of organs involved in metastasis, and histological subtypes. When evaluating the number of metastatic organs, the presence of multiple metastases was significantly associated with a negative change in ΔSMI, similar to the association observed with low muscle mass (p = 0.021).

### Evaluation of response to treatment

In the sarcopenic group, only 2 patients (7%) had an objective response to treatment, while 28 patients (93%) had no response. In the normal muscle masss group, 6 patients (30%) had an objective response to treatment, while 14 patients (70%) had no objective response. The objective response rate in the normal muscle mass group was found to be statistically significant (p = 0.027). When evaluated in terms of objective response, 24 patients (96%) in the ΔSMI-negative group did not achieve an objective response to treatment, whereas 18 patients (72%) in the ΔSMI-positive group also did not achieve an objective response (p = 0.021). The association of the presence or absence of low muscle mass and change in SMI with response to treatment is summarized in Table 1 and Table 2.

**Table 1.** Comparison of clinical characteristics and survival outcomes by baseline muscle mass status

Feature	Low buscle mass present (n = 30)	Low muscle mass absent (n = 20)	p-value
Patients, n (%)	30 (60)	20 (40)	-
Age, mean ± SD	60 ± 10.5	57.7 ± 9.9	0.382
Sex (men / women)	25 / 5	10 / 10	0.013*
IMDC score (favorable / intermediate / poor)	23 / 2 / 2	13 / 3 / 2	0.541
Objective treatment response (Yes / No)	2 / 28	6 / 14	0.027*
Overall Survival, Months (95% CI)	20 (8.1–31.9)	NR	< 0.001*
Progression-Free Survival, Months (95% CI)	8.8 (5.7–11.9)	30.2 (13.1–47.4)	0.004*

* Statistically significant p-values are marked with an asterisk. Mann–Whitney U, Chi-square, and log-rank tests were used where appropriate.

1 CI = confidence interval; HR = hazard ratio; IMDC = International Metastatic Renal Cell Carcinoma Database Consortium; ΔSMI = change in skeletal muscle index

**Table 2.** Association of change in skeletal muscle index with clinical and survival outcomes

Variable	ΔSMI Negative (n = 25)	ΔSMI Positive (n = 25)	p-value
Age, mean ± SD	61.0 ± 10.1	57.1 ± 10.1	0.180
Sex (men/ women)	18 / 7	17 / 8	0.758
IMDC score (favorable / intermediate / poor)	18 / 1 /3	18 / 4 / 1	0.249
Presence of multiple metastases, n (%)	19 (76)	11 (44)	0.021*
Baseline low muscle mass, n (%)	19 (76)	11 (44)	0.021*
Objective treatment response (Yes / No)	1 / 24	7 / 18	0.021*
Overall survival, months (95% CI)	15.8 (0–37.0)	NR	0.027*
Progression-free survival, months (95% CI)	8.1 (1.6–14.6)	30.2 (11.5–49.0)	0.005*

* Low muscle mass defined as SMI < 52.4 cm^2^ / m^2^ in men and < 38.5 cm^2^/m^2^ in women. Statistically significant p-values are marked with an asterisk. Mann–Whitney U, chi-square, and log-rank tests were used where appropriate.

1 CI = confidence ınterval; IMDC = International Metastatic Renal Cell Carcinoma Database Consortium; NR = not reached; ΔSMI = change in skeletal muscle ındex

### Survival analysis

**FIGURE 3.**
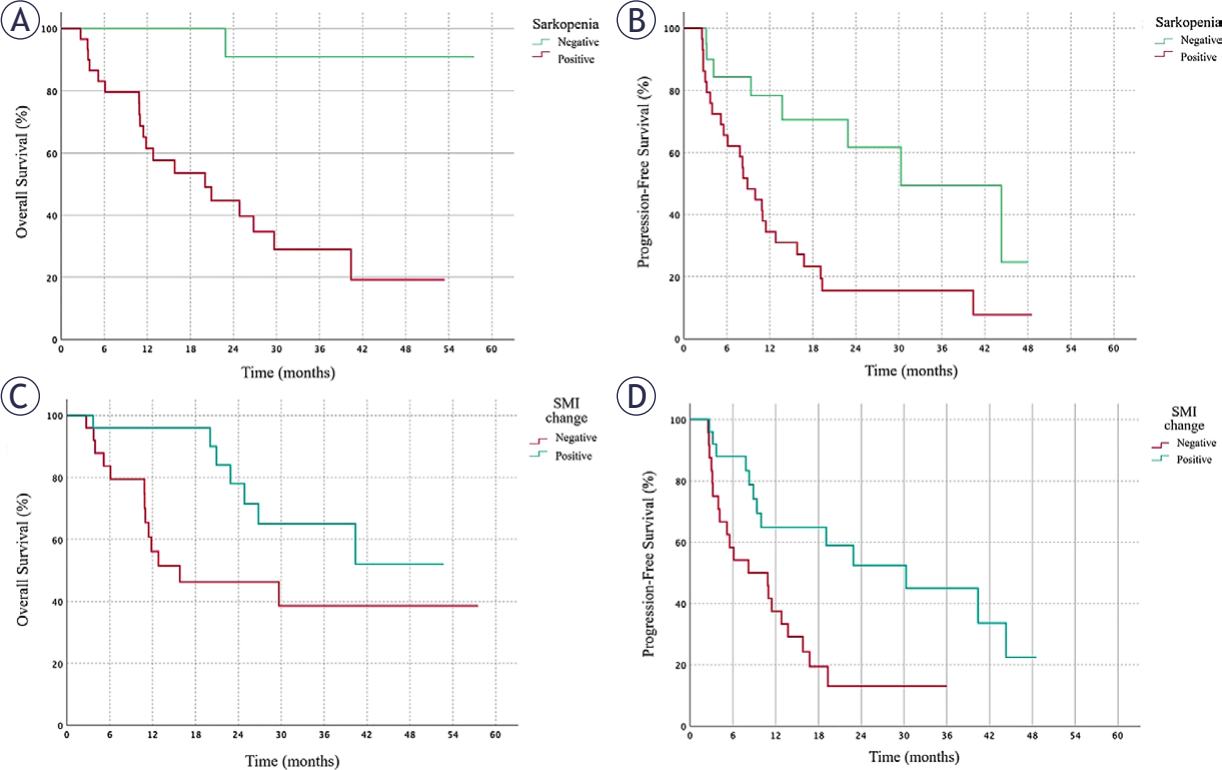
Kaplan-Meier survival curves illustrating overall survival (OS) and progression-free survival (PFS) in patients with metastatic renal cell carcinoma stratified by muscle mass status and skeletal muscle index (SMI) change. **(A)** OS was significantly shorter in patients with low muscle mass compared to those normal muscle mass group (HR: 35.00; 95% CI: 3.22-381.69; p = 0.003). **(B)** PFS was significantly shorter in patients with low muscle mass than in normal muscle mass group (HR: 12.50; 95% CI: 2.10-73.91; p = 0.004). **(C)** Patients with a negative ΔSMI had significantly reduced OS compared to those with a positive ΔSMI (HR: 6.10; 95% CI: 1.46-25.47; p = 0.013). **(D)** A negative ΔSMI was associated with significantly shorter PFS compared to a positive ΔSMI (HR: 4.50; 95% CI: 1.15-17.65; p = 0.031) CI = confidence interval; HR = hazard ratio; HU = Hounsfield unit; OS = overall survival; PFS = progression-free survival; ΔSMI = change in skeletal muscle index

The median OS for the entire group was 40.3 months (95% CI, 16.9–63.8), and the median PFS was 12.8 months (95% CI, 6.9–18.6). While the median survival could not be reached in the normal muscle mass group, the median OS in the low muscle mass group was 20 months (p < 0.001) (Figure 3A). The PFS for the low muscle mass group was 8.8 months, while it was 30.2 months in the normal muscle mass group, which was statistically significantly higher (p = 0.004) (Figure 3B).

The association of low muscle mass with clinical features and survival is summarized in Table 1. Furthermore, OS and PFS were significantly higher in the ΔSMI-positive group compared to the ΔSMI-negative group (p = 0.027 and p = 0.05, respectively) (Figure 3C–D). The association of changes in muscle mass with survival is summarized in Table 2.

### Univariate and multivariate analysis

Univariate and multivariate analyses were performed to assess the associations of OS and PFS with age, sex, histologic type, presence or absence of low muscle mass, ΔSMI, IMDC risk group, number of metastases and metastasis site.

In the univariate Cox regression analysis, low muscle mass prior to nivolumab treatment (hazard ratio [HR]: 17.50; 95% CI: 2.53–121.03; p = 0.005), a negative change in ΔSMI (HR: 2.72; 95% CI: 1.0–86.88; p = 0.034), and the presence of multiple organ metastases (HR: 3.11; 95% CI: 1.12–8.64; p = 0.029) were found to be significant prognostic factors for OS.

In the multivariate analysis, low muscle mass remained an independent predictor of mortality, with affected patients exhibiting significantly shorter OS compared to normal muscle mass individuals (HR: 35.00; 95% CI: 3.22–381.69; p = 0.003). Likewise, a negative ΔSMI was associated with increased mortality (HR: 6.10; 95% CI: 1.46–25.47; p = 0.013), and multiorgan metastases showed a borderline association with poorer OS (HR: 5.21; 95% CI: 1.00–27.10; p = 0.050) (Table 3).

**Table 3.** Cox regression analysis for overall survival

Variable	Univariate HR (95% CI)	p-value	Multivariate HR (95% CI)	p-value
Age	1.01 (0.96–1.05)	0.594	0.98 (0.92–1.05)	0.713
Sex (female *vs*. male)	0.89 (0.34–2.33)	0.818	0.86 (0.22–3.25)	0.825
Low muscle masss (present *vs*. absent)	17.50 (2.33–131.10)	0.005*	35.00 (3.22–381.69)	0.003*
ΔSMI (negative *vs*. positive)	2.72 (1.07–6.87)	0.034*	6.10 (1.46–25.47)	0.013*
IMDC score (intermediate/poor *vs*. favorable)	0.67 (0.15–2.94)	0.600	1.57 (0.26–9.38)	0.621
Multiple metastases (present *vs*. absent)	3.11 (1.12–8.64)	0.029*	5.21 (1.00–27.10)	0.050*
Histologic subtype (non-clear cell *vs*. clear cell)	0.85 (0.11–6.40)	0.877	0.68 (0.04–9.56)	0.779
Lymph node metastasis (present *vs*. absent)	0.82 (0.34–1.98)	0.665	0.31 (0.07–1.22)	0.094
Lung metastasis (present *vs*. absent)	1.26 (0.51–3.09)	0.614	0.27 (0.05–1.39)	0.118
Liver metastasis (present *vs*. absent)	1.78 (0.71–4.49)	0.216	0.94 (0.24–3.67)	0.940

* Statistically significant p-values are marked with an asterisk.

1 CI = confidence interval; HR = hazard ratio; IMDC = International Metastatic Renal Cell Carcinoma Database Consortium; ΔSMI = change in skeletal muscle index

In the univariate analysis, low muscle mass (HR: 5.80; 95% CI: 1.80–18.60; p = 0.003), negative ΔSMI (HR: 3.40; 95% CI: 1.20–9.70; p = 0.027), and multiple organ metastases (HR: 2.88; 95% CI: 1.057.92; p = 0.038) were identified as significant predictors of shorter PFS.

Multivariate analysis revealed that low muscle mass remained an independent predictor of disease progression (HR: 12.50; 95% CI: 2.10–73.91; p = 0.004), as did negative ΔSMI (HR: 4.50; 95% CI: 1.15–17.65; p = 0.031). The association between multiorgan metastases and PFS remained borderline significant (HR: 3.80; 95% CI: 0.98–14.80; p = 0.053) (Table 4).

**Table 4.** Cox regression analysis for progression-free survival

Variable	Univariate HR (95% CI)	p-value	Multivariate HR (95% CI)	p-value
Age	0.99 (0.96–1.03)	0.647	0.98 (0.93–1.03)	0.437
Sex (female *vs*. male)	0.87 (0.38–2.00)	0.739	1.00 (0.33–3.05)	0.994
Low muscle mass (present *vs*. absent)	4.17 (1.91–9.10)	< 0.001*	4.98 (1.99–12.42)	< 0.001*
ΔSMI (Negative *vs*. Positive)	3.52 (1.50–8.26)	0.004*	6.42 (2.18–18.91)	0.001*
IMDC score (intermediate/poor *vs*. favorable)	1.13 (0.33–3.84)	0.849	2.12 (0.48–9.30)	0.321
Multiple metastases (present *vs*. absent)	2.53 (1.07–5.97)	0.035*	1.97 (0.59–6.63)	0.267
Histologic subtype (non-clear cell *vs*. clear cell)	0.95 (0.18–5.01)	0.950	1.39 (0.15–12.92)	0.771
Lymph node metastasis (present *vs*. absent)	0.76 (0.34–1.70)	0.510	0.52 (0.17–1.63)	0.264
Lung metastasis (present *vs*. absent)	1.18 (0.52–2.67)	0.692	0.59 (0.18–1.89)	0.374
Liver metastasis (present *vs*. absent)	1.33 (0.62–2.88)	0.466	0.89 (0.30–2.66)	0.839

* Statistically significant p-values are marked with an asterisk.

1 CI = confidence interval; HR = hazard ratio; IMDC = International Metastatic Renal Cell Carcinoma Database Consortium; ΔSMI = change in skeletal muscle index

## Discussion

This study demonstrated that both baseline low muscle mass and negative change in SMI during nivolumab therapy were strongly associated with reduced treatment efficacy, shorter PFS and lower OS in patients with mRCC. Additionally, both low muscle mass and a negative ΔSMI were significantly correlated with a higher incidence of multiorgan metastases. These findings underscore the prognostic importance of skeletal muscle status in the context of immunotherapy.

While the association between low muscle mass and poor outcomes in mRCC has been previously established, our study provides several novel insights. First, we demonstrate that dynamic changes in muscle mass during nivolumab treatment (ΔSMI) serve as independent predictors of both survival outcomes and treatment response, with patients experiencing muscle mass decline showing significantly worse outcomes even when baseline muscle status is considered. This dynamic assessment approach has been underexplored in the immunotherapy literature. Second, our use of iRE-CIST criteria for response evaluation in the context of muscle mass assessment addresses the unique challenges of immunotherapy response patterns, including pseudoprogression, which may confound traditional response assessments. Third, our findings specifically validate the prognostic utility of muscle mass assessment in nivolumab-treated patients, contributing to the growing evidence base for precision medicine approaches in immunotherapy selection and monitoring.

Our results are in line with a recent meta-analysis, which reported significantly worse OS in sarcopenic patients compared to non-sarcopenic individuals across both localized and mRCC populations.^19^ While Herrmann *et al*. did not find a statistically significant OS difference, other studies such as those by Fukushima *et al*. and Sharma *et al*. demonstrated that sarcopenia is an independent predictor of poor survival.^10,20,21^ Our cohort further supports these findings, with significantly shorter median OS in sarcopenic patients. Furthermore, patients who developed a positive ΔSMI during the course of treatment were found to have longer OS compared to patients who experienced a decrease in muscle mass.

The PFS findings in our study are also consistent with the existing literature. Ueki *et al*. reported a median PFS of 8.3 months in sarcopenic patients and 48.4 months in non-sarcopenic patients in their study in mRCC patients using nivolumab.^22^ In our cohort, median PFS was similarly reduced in sarcopenic patients and those with negative ΔSMI, emphasizing the potential value of dynamic muscle assessment during immunotherapy.

Another clinically important observation was the significantly lower objective response rate in sarcopenic patients and those with reduced SMI. These findings echo those of Ishihara *et al*. who reported reduced treatment response in patients with progressive muscle wasting during targeted therapy.^23^ While their study is relevant to sunitinib, the consistency in our nivolumab-treated cohort suggests a broader association between low muscle mass and therapeutic resistance.

Importantly, we observed that patients with baseline low muscle mass were more likely to experience additional muscle loss during treatment. This observation, consistent with previous findings in sunitinib-treated patients, may reflect a compounding effect of pre-existing low muscle mass and treatment-related catabolism. Additionally, patients with negative ΔSMI were significantly older, suggesting that age-related sarcopenia and tumor-related cachexia may exert synergistic effects in promoting muscle degradation.^23^

Skeletal muscle acts as a dynamic endocrine and immunomodulatory organ by releasing myokines that influence immune responses, including the activation and regulation of cytotoxic T lymphocytes.^24^ Sarcopenia, compromises immune surveillance and reduces therapeutic response to immune checkpoint inhibitors.^25,26^ Furthermore, chronic systemic inflammation, commonly observed in sarcopenic individuals, contributes to the development of an immunosuppressive tumor micro-environment, further compromising treatment efficacy.^27^

Generally, sarcopenia has emerged as a promising imaging-derived biomarker reflecting host physiology, immune competence and systemic inflammation.^28^ Unlike molecular biomarkers such as PD-L1 expression and tumor mutation burden, which have shown limited predictive utility in immunotherapy, muscle status provides a comprehensive, patient-level insight into biological reserve and treatment tolerance. Several systemic inflammatory markers such as C-reactive protein and neutrophil/lymphocyte ratio have been investigated as prognostic indicators.^22,23^ However, these are non-specific and are vulnerable to confounding by infections or treatment-related toxicities.

Muscle mass can be objectively and reproducibly assessed by imaging modalities such as CT, making it a practical tool for the assessment of low muscle mass in oncology. However, although CT-based quantitative muscle mass measurement is a validated and reproducible method to assess amount of muscle mass, it does not capture muscle strength or physical performance. Accordingly, integrating imaging assessments with functional assessments such as grip strength or walking speed would improve the clinical relevance of measuring sarcopenia.

Recent studies have emphasized the importance of muscle quality, particularly skeletal muscle radiodensity, as an additional determinant of treatment outcomes. Low muscle attenuation values, indicative of increased fat infiltration (myosteatosis), have been associated with worse clinical outcomes in patients receiving immunotherapy.^29,30^ However, variability in CT protocols, including the use of intravenous contrast agents, may limit the consistency of radiodensity measurements between centers.

This study has several limitations. First, its retrospective and single-center design may affect the generalizability of our findings. Second, while our sample size (n = 50) is comparable to other nivolumab studies, it limits the statistical power for subgroup analyses. Additionally, it is important to acknowledge that our study assessed only muscle quantity (mass) through CT-based SMI measurements, which represents only one component of the comprehensive sarcopenia assessment as defined by EWGSOP2. True sarcopenia diagnosis requires assessment of muscle strength and potentially physical performance in addition to muscle mass. Therefore, our findings specifically relate to low muscle mass rather than confirmed sarcopenia. While muscle mass serves as a practical and widely available imaging biomarker, future studies incorporating functional assessments would provide a more comprehensive evaluation of muscle status and its relationship to treatment outcomes.

Despite these limitations, the primary outcomes in our study were robust, and both low muscle mass and ΔSMI remained independent predictors of OS and PFS in multivariate analyses. Our findings support the integration of skeletal muscle evaluation into routine oncologic assessment for patients receiving immunotherapy. Future prospective studies incorporating nutritional and rehabilitative interventions are warranted to validate and expand these observations.

## Conclusions

This study demonstrates that both baseline low muscle mass and treatment-related muscle mass decline are associated with poorer treatment response, shorter PFS, and lower OS in mRCC patients receiving nivolumab. The dynamic assessment of muscle mass changes during treatment provides additional prognostic information beyond baseline measurements. Routine CT-based assessment of muscle mass can serve as a practical imaging biomarker to complement existing prognostic tools, though integration with functional assessments would enhance its clinical utility. Larger-scale prospective studies incorporating both structural and functional muscle parameters are needed to validate these findings and explore interventional strategies.
